# Uniform-Sized Indium Quantum Dots Grown on the Surface of an InGaN Epitaxial Layer by a Two-Step Cooling Process

**DOI:** 10.1186/s11671-019-3095-7

**Published:** 2019-08-16

**Authors:** Shuangtao Liu, Jing Yang, Degang Zhao, Desheng Jiang, Jianjun Zhu, Feng Liang, Ping Chen, Zongshun Liu, Yao Xing, Liyuan Peng, Liqun Zhang

**Affiliations:** 1 State Key Laboratory of Integrated Optoelectronics, Institute of Semiconductors, Chinese Academy of Sciences, Beijing, 100083 China; 2 Center of Materials Science and Optoelectronics Engineering, University of Chinese Academy of Sciences, Beijing, 100049 China; 3 Suzhou Institute of Nano-tech and Nano-bionics, Chinese Academy of Sciences, Suzhou, 215123 China

**Keywords:** Single InGaN layer, In quantum dots, In-rich layer

## Abstract

A new method to grow Indium quantum dots (In QDs) on the surface of an epitaxial InGaN layer by MOCVD is proposed. Uniform-sized In quantum dots have been found to form on the surface of an InGaN layer when a two-step cooling process is taken. Through analyzing, we found that the formation of In QDs on the surface is due to the reaction between the surface In-rich layer and the carrier gas H_2_ at the lower temperature period in the two-step cooling process. At the same time, as the density of In QDs is closely dependent on the surface In-rich layer, this provides us a way to study the surface property of the InGaN layer directly.

## Introduction

Recent years, (Al, In, Ga)N-based materials have attracted a great deal of attention due to their successful application in a light-emitting device (LED) and laser diode (LD) [1–5]. InGaN has high absorption, broad spectral coverage, and radiation hardness and it is always used as an active material for the fabrication of optoelectronic devices. However, it is a big challenge to grow high-quality InGaN materials, due to a number of problems. For instance, the large difference in lattice constant between InN and GaN results in a solid-phase miscibility gap [6, 7]. The relatively high vapor pressure of InN compared to GaN leads to a low indium incorporation in the InGaN alloy [8]. In addition, a large difference in formation enthalpies for InN and GaN causes a strong indium surface segregation on the growth front [9]. On the other hand, in the process of InGaN-layer growth, there always exists an In-rich layer on the surface due to the pulling effect, which will affect the quality of subsequent layer of InGaN/GaN multiple quantum well (MQW) by introducing indium atoms in subsequent GaN layer growth [10]. For getting the high performance of InGaN-based optoelectronic devices, we have to overcome all these obstacles. During the research, the growth of a single InGaN layer is always used to optimize the growth parameter of InGaN/GaN MQW. In this paper, we occasionally found uniform-size In quantum dots form on the surface of the single InGaN layer when taking a two-step cooling down process which replaces the regular one-step cooling down process after the growth of the single InGaN layer. Through analysis, we found that the formation of In QDs is related to the In-rich layer existing on the surface of the InGaN layer, and that provides us a way to study the surface In-rich layer directly.

## Experiment

**Fig. 1:**
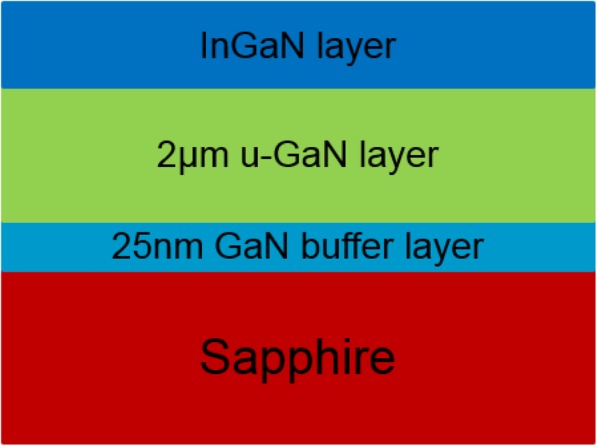
The structure schematic of the single InGaN layer grown on a GaN template by using a sapphire substrate

Samples used in this study were single InGaN layers grown on c-plane sapphire substrate by an AIXTRON 6 × 2 in close-coupled showerhead reactor metalorganic chemical deposition (MOCVD) in a N_2_ atmosphere. Trimethylgallium (TMGa), trimethylindium (TMIn), and ammonia (NH_3_) are used as Ga, In, and N source precursors, respectively. The single InGaN layers with a thickness below 60 nm and the In content of less than 15% were grown on a 2-μm-thick unintentional doping GaN layer after a 25-nm GaN buffer layer grown on a sapphire substrate. The structure schematic is shown in Fig. 1. H_2_ and N_2_ are used as a carrier gas in different periods of the growth process. Conventionally, the N_2_ is used as carrier gas in the process of InGaN layer growth because H_2_ has a corrosive effect on InGaN layer which will largely decrease the efficiency of In incorporation [11, 12]. However, H_2_ as carrier gas can greatly improve the surface mobility of atoms and reduce the impurity concentration. Therefore, H_2_ is preferred to use as a carrier gas during the growth of the GaN layer to improve the crystal quality. Generally, when the growth of the InGaN layer is finished, the temperature was directly decreased to room temperature in a nitrogen atmosphere. Here, we call such a process as “one step cooling”. In this paper, a so-called two-step cooling process has been taken on after the InGaN layer growth, in which the temperature was lowered to 400 °C in a nitrogen atmosphere at first cooling period, and subsequently it is reduced to room temperature in a H_2_ atmosphere. High-resolution X-ray diffraction (XRD), atomic force microscopy (AFM), scanning electron microscope (SEM), and energy-dispersive spectrometer (EDS) are used to characterize the InGaN samples.

## Result and Discussion

**Fig. 2:**
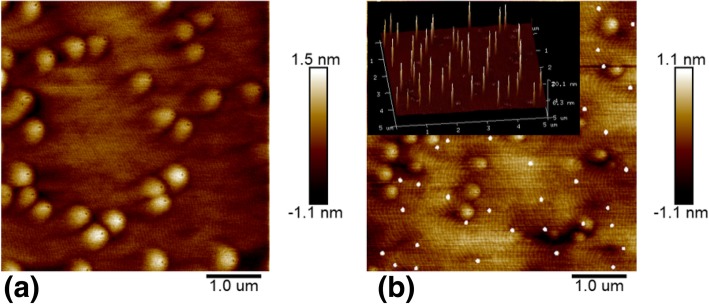
AFM surface topography of **a** InGaN sample with one-step cooling and **b** InGaN sample with two-step cooling process where the inset is a 3D diagram of surface

An abnormal surface topography of the InGaN layer has been found when grown on a single InGaN layer on the GaN template with a two-step cooling down process. The AFM surface morphology of the InGaN samples with one-step cooling and two-step cooling is respectively shown in Fig. 2a and b. Figure 2a shows a typical surface morphology of InGaN epilayer, from which we can find that the InGaN layer has a clear steps flow 2D growth. At the same time, there exists many 3D islands on the sample surface which is attributed to relate with screw dislocation lines in the GaN layer. It can also be seen that there is a dark dot on the top of each 3D island which has been proven to be the v-pit formed along the screw dislocation [13, 14]. In difference from Fig. 2a, except the step flow surface and 3D islands, there are also many uniform-sized quantum dots (the small white dots in the picture) on Fig. 2b. Combined with the inset of Fig. 2b on the left upper corner which is a 3D diagram of the surface, we can get the average size for these quantum dots is about 100 nm × 100 nm, the average height is about 20 nm, and the density is around 1.6 × 10^8^ cm^−2^. It is noted that the main difference between these two different ways of sample cooling is that H_2_ is used as carrier gas instead of N_2_ in the second cooling period of the two-step cooling process. Therefore, the formation of quantum dots on surface of the InGaN layer is related to carrier gas H_2_ in the low-temperature cooling process.

**Fig. 3:**
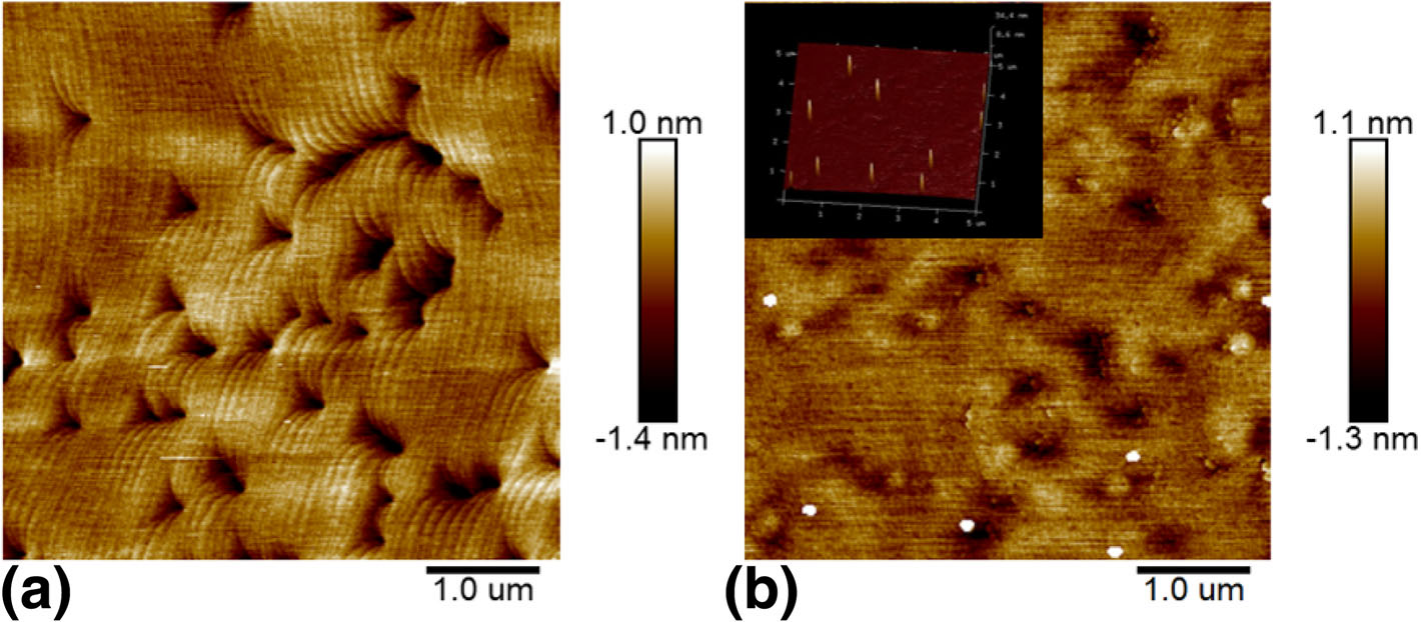
AFM surface topography of samples with two-step cooling. **a** GaN layer. **b** InGaN layer with In content 1%

To understand how this happens when two-step cooling process is taken on InGaN samples, we make a two-step cooling experiment on GaN instead of InGaN. This GaN layer was grown on a condition the same with the single InGaN layer samples which studied in this work, i.e., at a relatively lower growth temperature of 740 °C and N_2_ is used as a carrier gas. The AFM surface topography of the GaN layer after a two-step cooling process is shown in Fig. 3a. We can find that there are no any quantum dots that exist on the surface and the surface is just the same as one-step cooling MOCVD-grown GaN layer samples as have been widely reported [15]. In addition, we have checked what will happen when the two-step cooling process is used for an InGaN layer sample with very low In content in which the In content is only 1%. Fig. 3b shows the AFM surface morphology of this InGaN sample with low In content after a two-step cooling process. It is found that the density of quantum dots has a very large decrease compared to the InGaN sample with high In content mentioned above which the In content is about 13%. These results mean that the formation of quantum dots on the surface of the InGaN layer in the two-step cooling process is correlated to the In atom, and these quantum dots may be one of In state which is formed due to the reaction between the InGaN layer and carrier gas H_2_ in the process of second cooling.

**Fig. 4:**
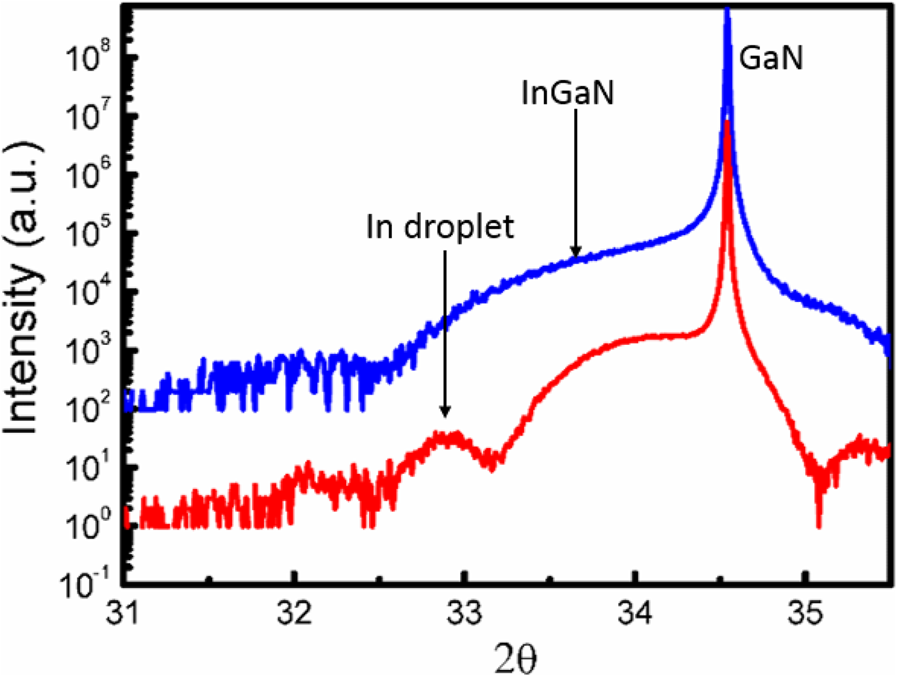
The XRD spectra for sample with two steps cooling (red line) and one step cooling (blue line)

**Fig. 5:**
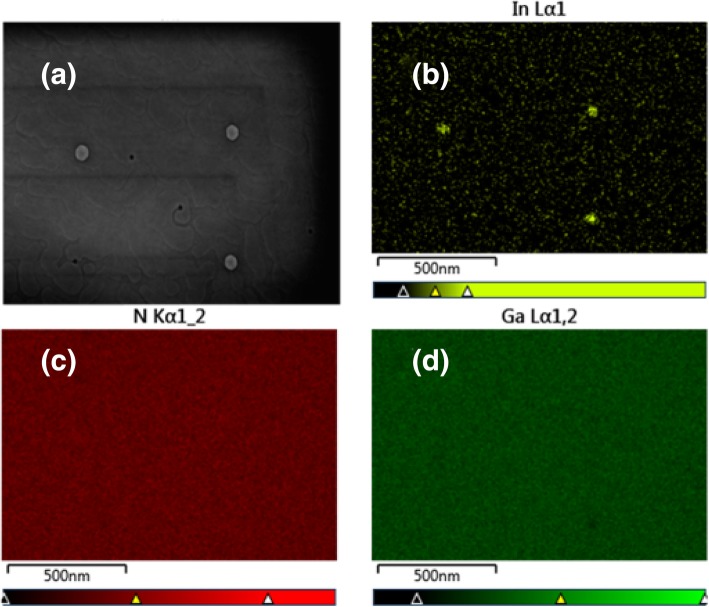
**a** The SEM surface photography for the sample with a two-step cooling process, **b**–**d** is the EDS element analysis result for atom In, N, and Ga, respectively

Previous studies have shown that there exist three different states of In atoms in the process of InGaN layer growth [16, 17]. The first one is the In atoms in InGaN alloy crystal lattice, which is the main part and the target product of InGaN layer growth; the second one is the InN alloy which always appears on the surface of the InGaN layer; and the third one is the In droplet which may appear in some special growth condition. Different states of the In atom can be distinguished by XRD spectra as they have different diffraction peaks in the ω/2θ scan mode. To determine which In state these dots belong to, XRD measurement has been taken for the InGaN samples with one-step or two-step cooling processes. Figure 4 shows the typical XRD ω/2θ spectra for samples with two-step cooling (red line) and one-step cooling (blue line). Three characteristic peaks were found for the samples with two-step cooling while there are only two characteristic peaks for samples with one-step cooling. So the extra characteristic peak for the samples with two-step cooling may represent the quantum dots that form on the surface. The two characteristic peaks which commonly exist in these samples are located at around 33.5° and 34.5°. They belong to InGaN and GaN crystal, respectively. While the extra characteristic peak located at the 2θ = 32.8° has been verified to come from the In droplet [16, 17]. So we can make a conclusion that the quantum dots we observed on the InGaN surface with the two-step cooling process is the In droplet. In order to confirm this conclusion, SEM measurement has been taken on the samples with a two-step cooling process, the result is shown in Fig. 5a, from which we can also find the dots exist on the surface. To identify these dots, EDS has been taken on the same place, Fig. 5b–d show the surface atom distribution for In, Ga, and N, respectively, from which we can get that the atom of Ga and N are evenly distributed on the surface, while the In atoms concentration on the dots is much larger than its surrounding, which represent that these dots are In rich. So combined with the above analysis, we can conclude that carrier gas H_2_ can react with the InGaN layer in the low temperature and form In droplets on the surface.

**Fig. 6:**
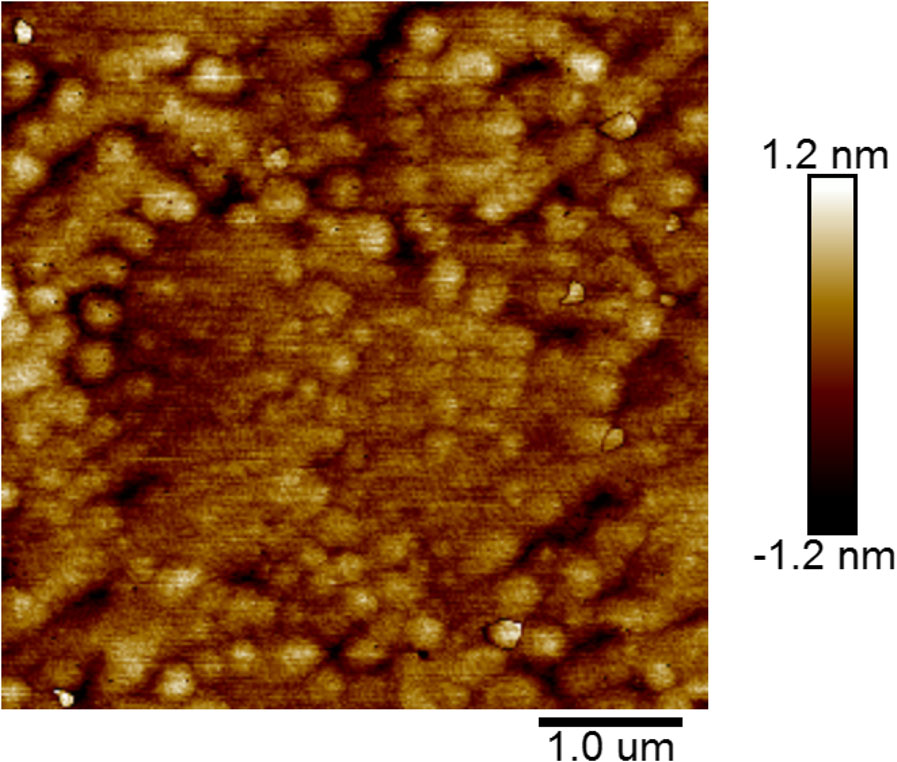
The AFM surface topography of the InGaN layer with a thermal annealing before the two-step cooling.

It has been reported that there is often an In-rich layer formed on the surface of the InGaN epitaxial layer due to the surface pulling effect [10]. In our experiment, the samples were directly cooled down to room temperature after the growth of a single InGaN layer, which means that the In-rich surface layer still exists on the surface in the cooling process. Therefore, there are two possibilities of the origin of In atom for the In QDs we observed: the InGaN layer and In-rich surface layer, respectively. To know what is the origin of the In atom of the In quantum dots and how the reaction happens, further experiments have been carried out. It is known that an annealing process can wipe the In-rich layer off [18]. Therefore, we take a thermal annealing process for the InGaN samples before the two-step cooling process. The time of the thermal annealing process is set as 60 s, and the temperature is about 800 °C which is 60° higher than the growth temperature of the InGaN layer. The AFM topography of the InGaN sample with an annealing process before the two-step cooling process is shown in Fig. 6, obviously there is no quantum dots on the surface. However, compared to Fig. 2a, a big change of the surface topography can be found, the surface becomes more rough. The lack of In quantum dots on the surface shows that the InGaN layer would not form In QDs with H_2_ in the second cooling process, as thermal annealing can only wipe the In-rich layer out. So the formation of In quantum dot on the surface of the InGaN layer in the two-step cooling process is due to the reaction between In-rich surface layer and carrier gas H_2_ in the low-temperature range.

The corrosive effect of H_2_ on the growth of the InGaN layer has been largely reported. It is known that H_2_ can hinder the In atoms to incorporate into the lattice [19, 20], and that is the main reason why conventionally only N_2_ is used as a carrier gas in the MOCVD growth of InGaN layers. The corrosive effect of H_2_ on the InGaN layer can be seen as a reverse process of the InGaN layer growth, which can be expressed by the following chemical reaction:

$$ {3\mathrm{H}}_2+2\mathrm{In}\mathrm{N}\to 2\mathrm{In}+{2\mathrm{NH}}_3 $$

The formation of In quantum dots in the two-step cooling process can be seen as a kind of corrosive effect, but there are some differences between high temperature (to grow InGaN with H_2_) and low temperature (to cool InGaN and form In QDs with H_2_). At high temperature, the In atom formed by the corrosive effect on the surface have enough energy to escape across the surface boundary layer, thus decreasing the efficiency of In incorporation. However, at low temperature (below 400 °C), our results reveal that H_2_ only has a corrosive effect on highly In-contained surface layer as this layer is more unstable than the InGaN layer. On the other hand, as the temperature is below 400 °C, the In atoms form on the surface can much less escape across the surface boundary timely, so they will migrate on the sample surface and then form In drops on the surface.

**Fig. 7:**
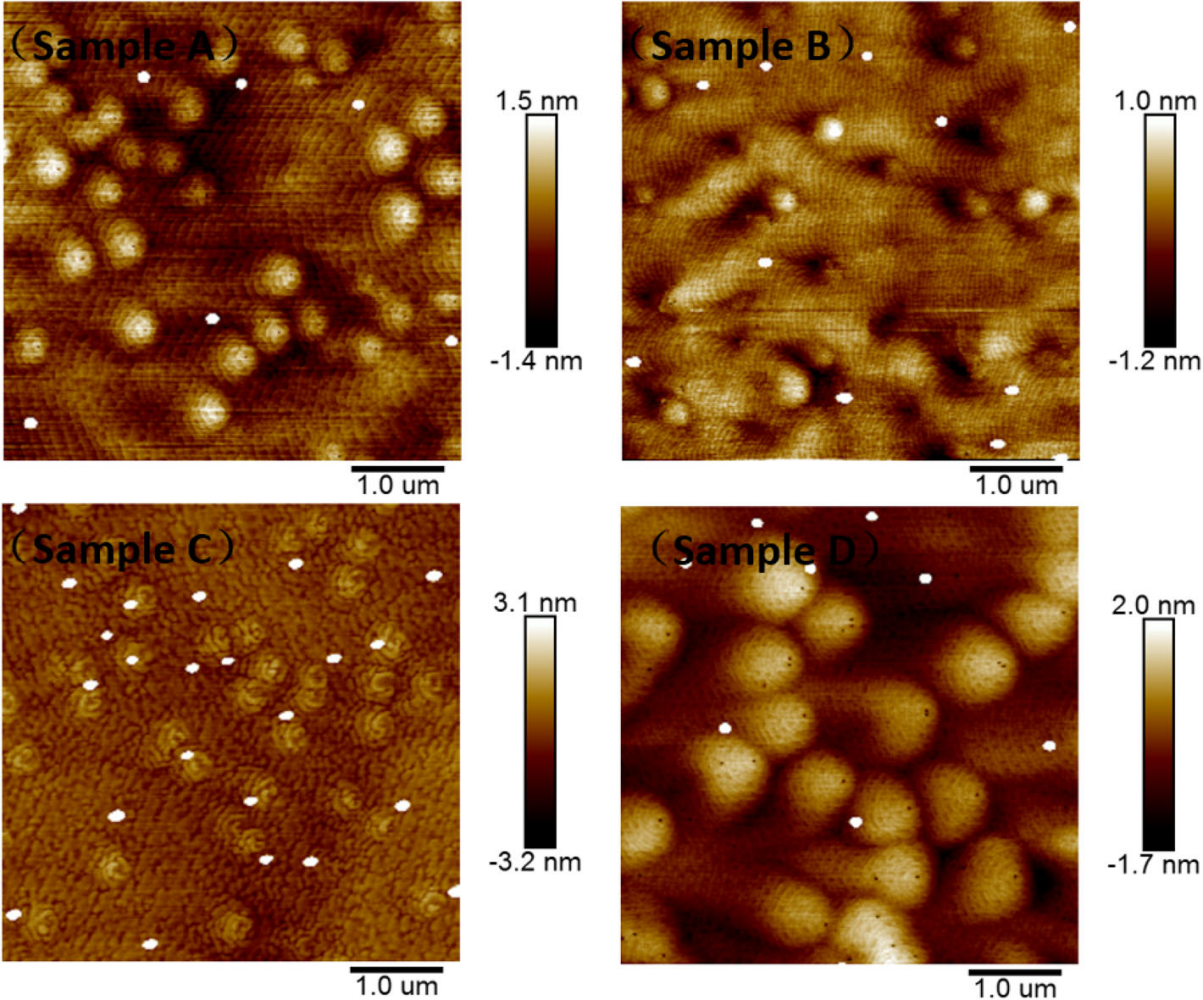
The AFM surface topography of InGaN layers **a**, **b**, **c**, and **d** with different In content and thickness (see text)

In our study, as the formation of In QDs is related to the high In contained layer on the InGaN surface, so they can directly provide some information about the surface In-rich layer of InGaN. The InGaN layer samples with different In content and thickness are used to take two-step cooling treatment experiments. Figure 7 shows the surface topography of InGaN layers with different In content and thickness. The In content is 7.65%, 8.45%, and 9.6%, respectively, for samples A, B, and C, and the thickness of the InGaN layer for these three samples is almost the same (about 13.4 nm). From the AFM surface morphologies of sample A, B, and C, we can find that the density of In QDs increases with In content in the InGaN layer. And the density of QDs is about 2.4 × 10^7^ cm^−3^, 4.8 × 10^7^ cm^−3^, and 9.2 × 10^7^ cm^−3^ respectively for samples A, B, and C, while the size of QDs for these samples is almost the same. On the other hand, sample D has the same In content with sample A (7.65%), while its layer thickness is about 41 nm. Compare the surface for two samples of A and D, it is clear that the density of In quantum dots increases when the thickness of the InGaN layer increases. From our analysis mentioned above, it is learned that the formation of these In quantum dots is caused by the reaction of H_2_ with the in-rich surface layer at low temperature. So the changing tendency of In QDs density can reflect the state of the In-rich surface layer, i.e., the unstable In atoms existing in the In-rich surface layer, they increase along with the increase of the In content and thickness of the InGaN layer. This result is consistent with previous theoretical studies which have shown that the In content of the In-rich surface layer is related to the In content and the thickness of the InGaN layers [21]. This also proves that the formation mechanism of In quantum dots is indeed related to the surface of the In-rich layer.

## Conclusion

In this paper, we have introduced a new method to obtain uniform-sized In QDs on the surface of an InGaN layer. We found uniform-sized In QDs form on the surface of an InGaN layer when taking a two-step cooling process on it. Through a detailed analysis, we found that the formation of In quantum dots on the surface is due to the reaction between surface In-rich layer and carrier gas H_2_ at a low temperature. At the same time, our experiments reveal that H_2_ only has a corrosive effect on In-rich surface layer when the temperature is lower than 400 °C, and this corrosive process will form In QDs on the surface. On the other hand, as the formation of In QDs is closely related to the In-rich layer on the surface, such a process can provide us a way to study the property of this layer directly.

## Method

The aim of this paper is to introduce a new method to get uniformed-size In QDs on the surface of the InGaN layer. Samples used in this study were grown by an AIXTRON 6 × 2 in close-coupled showerhead reactor metalorganic chemical deposition (MOCVD). High-resolution X-ray diffraction (XRD), atomic force microscopy (AFM), scanning electron microscope (SEM), and energy-dispersive spectrometer (EDS) are used to characterize the InGaN samples. All the participants of this study are the scientist from the University of Chinese Academy of Sciences of China.

## Data Availability

The datasets used and/or analyzed during the current study are available from the corresponding author on reasonable request.

## Abbreviations

AFM: Atomic force microscopy
EDS: Energy dispersive spectrometer
GaN: Gallium nitride
In QDs: Indium quantum dots
InGaN: Indium gallium nitride
InN: Indium nitride
LD: Laser diode
LED: Light-emitting device
MOCVD: Metalorganic chemical deposition
MQW: Multiple quantum well
NH_3_: Ammonia
QDs: Quantum dots
SEM: Scanning electron microscope
TMGa: Trimethylgallium
TMIn: Trimethylindium
XRD: X-ray diffraction
